# What Can Policy-Makers Get Out of Systems Thinking? Policy Partners’ Experiences of a Systems-Focused Research Collaboration in Preventive Health

**DOI:** 10.15171/ijhpm.2019.86

**Published:** 2019-11-03

**Authors:** Abby Haynes, Kate Garvey, Seanna Davidson, Andrew Milat

**Affiliations:** ^1^The Australian Prevention Partnership Centre, Sydney, NSW, Australia; ^2^Sydney School of Public Health, University of Sydney, Sydney, NSW, Australia.; ^3^Australia Public Health Services, Department of Health Tasmania, Hobart, TAS, Australia.; ^4^The Systems School, Melbourne, VIC, Australia.; ^5^Centre for Epidemiology and Evidence, NSW Ministry of Health, Sydney, NSW, Australia.

**Keywords:** Systems Thinking, Co-Production, Policy-Making, Capacity Development, Public Health

## Abstract

**Background:** There is increasing interest in using systems thinking to tackle ‘wicked’ policy problems in preventive health, but this can be challenging for policy-makers because the literature is amorphous and often highly theoretical. Little is known about how best to support health policy-makers to gain skills in understanding and applying systems thinking for policy action.

**Methods:** In-depth interviews were conducted with 18 policy-makers who are participating in an Australian research collaboration that uses a systems approach. Our aim was to explore factors that support policy-makers to use systems approaches, and to identify any impacts of systems thinking on policy thinking or action, including the pathways through which these impacts occurred.

**Results:** All 18 policy-makers agreed that systems thinking has merit but some questioned its practical policy utility. A small minority were confused about what systems thinking is or which approaches were being used in the collaboration. The majority were engaged with systems thinking and this group identified concrete impacts on their work. They reported using systems-focused research, ideas, tools and resources in policy work that were contributing to the development of practical methodologies for policy design, scaling up, implementation and evaluation; and to new prevention narratives. Importantly, systems thinking was helping some policy-makers to reconceptualise health problems and contexts, goals, potential policy solutions and methods. In short, they were changing how they think about preventive health.

**Conclusion:** These results show that researchers and policy-makers can put systems thinking into action as part of a research collaboration, and that this can result in discernible impacts on policy processes. In this case, action-oriented collaboration and capacity development over a 5-year period facilitated mutual learning and practical application. This indicates that policy-makers can get substantial applied value from systems thinking when they are involved in extended co-production processes that target policy impact and are supported by responsive capacity strategies.

## Background

Public health faces enormous operational and economic challenges as it wrestles with the burdens of an expanding and ageing population, rapidly changing technologies, and increasing demands for quality and efficiency.^1^ Yet the capacity of health policy agencies to respond effectively is constrained in several ways. First, by the *“wicked”* intractable nature of problems that have multiple causes and are characterised by uncertainty and conflicting values and views.^2-4^ Second, by the policy environment itself which is subject to multiple constraints. These include pressure from diverse stakeholders who have competing demands and expectations, politicised decision-making and a frequent need for rapid response.^5,6^ And third, by the highly complex open systems (such as communities, healthcare systems and nations) within which polices are implemented where leverage points are often outside of governments’ immediate sphere of influence.^7-11^ In short, public health policy is “...*embedded in intricate networks of physical, biological, ecological, technical, economic, social, political, and other relationships.”*^7^ Attempts to inform this work with research must take account of its complexity.^2,12-15^ Systems thinking is rapidly becoming key to this endeavor.^16,17^ As Kickbusch and Gleicher note:

*“Addressing wicked problems requires a high level of systems thinking. If there is a single lesson to be drawn from the first decade of the 21st century, it is that surprise, instability and extraordinary change will continue to be regular features of our lives.”* ^18^

Systems thinking in public health is a broad conceptual lens informed by a multi-disciplinary body of established theories, tools and methods.^17,19-21^ It posits that the world is comprised of systems—including health systems—which have interdependent parts that act synergistically and which constantly adapt in non-linear ways that can be resistant to ‘logical’ interventions. Systems thinking focuses on casual patterns rather than independent forces, and on root causes rather than symptoms.^22,23^ This, in turn, often indicates different leverage points from conventional methods, emphasises the need to respond to emergent developments rather than stick to an *a priori* formula,^11,12,19,25,26^ and places *“high value on understanding context and looking for connections between the parts, actors and processes of the system.”*^11^

These concepts have much in common with ecological models in community health and development that consider the social determinants of health,^26^ and resonate with the realities of policy-making which are frequently complex and tend to progress in recursive cycles rather than linear rational steps.^16^ But while systems thinking does incorporate these concepts it also goes much further *“... incorporating advances in fields such as organisational behavior , systems dynamics, emergence theory, and complexity theory.”*^26^ Notably, it focuses on a system’s relationships and conditions rather than on individual elements of the system.^27^ As Holmes and colleagues explain:

*“While social-ecological models have usefully reinforced the need for multiple levels of influence and multiple strategies ( eg , education, policy, media), they have been less effective in focusing attention on the interrelationships within and across levels and how interventions need to take these relationships into account in their design and implementation.”* ^28^

This amounts to a paradigm shift for public health which has traditionally been dominated by acute care and epidemiological models that focus on isolating independent actionable causes rather than viewing health and healthcare as long-term, evolving, contextually embedded and shaped by interconnected forces at micro, meso and macro levels.^11,29,30^ Indeed, the increasing focus on systems thinking is, in part, a response to the epidemic of chronic disease^18^ and an acknowledgement that interventions based on bio-medical models often take insufficient account of complexity and can therefore be a poor fit for the people they are trying to help and the contexts in which problems and solutions are located. Such interventions are less likely to effect desired change or to take account of the unintended consequences that interventions often trigger when introduced into systems that are composed of interdependent relationships.^31-34^

While there is increasing interest in using systems approaches in public health,^35^ its application presents considerable challenges for policy-makers, implementers and evaluators.^36^ Government processes and funding mechanisms tend to reinforce the status quo, and even when they understand the potential value of systems thinking they may not have the knowledge or skills to take action.^37,38^ Resources are available to support the practical use of systems thinking eg,^39-42^ but many lament a general emphasis on dense conceptual writing^43,44^ with an *“over-whelming focus on theory as opposed to practical application,”*^20^ and a tendency for policy-makers to receive abstract advice from systems scholars rather than concrete help.^45,46^

There is an increasing view that collaboration across government, disciplines, sectors and organisations is best suited to apply systems thinking in policy because it functions both as a means of informing policy and practice with systems science, and of working with the systems themselves.^12,13,34,47,48^ Collaboration—and co-production in particular—aims to harness the expertise of diverse stakeholders and disrupt the traditional *“linear model of research uptake [that] constructs evidence as an inert, apolitical entity to be implemented universally and unilaterally.”*^49^ Collaboration has been found to enhance the policy-relevance of research outputs, increase the value and uptake of research by policy partners, and to strengthen the capacity of members to undertake, share and use research effectively beyond the individual project via intellectual capital (knowledge) and social capital (relationships).^50-56^ Accordingly, there is a renewed focus globally on *“joined up”* governance and whole-of-society approaches that seek to improve the design, coordination and integration of policies for meeting shared goals.^18^ Such work will require systems skills if it is to be effective.^11,18,57,58^ Systems thinking has an increasing profile in policy dialogue but its application is still comparatively rare.^59^ Little is known about how policy-makers engage with systems thinking, or how the dynamics of collaboration can facilitate (or hinder) this engagement. Given that systems thinking has a pivotal role to play in tackling many of our most serious health problems, there is an increasingly pressing need to address these knowledge gaps.^11,16^

## Aims

This paper explores policy-makers’ experiences of engaging with systems thinking within a national cross-sector research collaboration in preventive health (described below). It describes their views about the value and impact of systems thinking, and the pathways through which it appears to be contributing to policy thinking and action. It addresses 4 research questions:

1. Why did policy-makers choose to participate in a systems-focused collaboration (what was in it for them)?
2. How are these policy partners experiencing systems thinking? (what’s working for them and what’s not?)
3. What is supporting policy partners to engage with systems thinking?
4. What value (if any) have policy partners found in systems thinking?

###  The Australian Prevention Partnership Centre

The Australian Prevention Partnership Centre (hereafter referred to as the *Prevention Centre* or *Centre*) was established in June 2013 as a National Health and Medical Research Council ‘Partnership Centre for Better Health.’^60^ Its goal is to develop a cross-sector collaboration to identify systems, strategies and structures for better decision-making in efforts to prevent lifestyle related chronic disease in Australia. This goal reflects recognition internationally that noncommunicable diseases are a serious and urgent population health problem^61^ which, despite their complex aetiology, are largely preventable.^62^

The Prevention Centre is a national partnership of researchers, policy-makers and practitioners. In its initial 5-year phase it included 31 Chief Investigators from academic institutions, health services and health policy agencies and its work expanded to include over 150 individuals implementing 40 research projects. During this period the Centre had resources (dollars and in-kind) of A$22.6 million provided by the National Health and Medical Research Council, Australia’s national department of health, two state/territory departments of health and a national private health insurer.

The Centre has an explicit commitment to co-producing practical systems-informed research, tools, resources and methods for tackling chronic disease prevention which can *“provide a way to examine complex problems, taking into account the bigger picture and context of those problems.”*^63^ Three primary models are used to ‘infuse’ systems thinking. First, scholarly research projects that are informed by systems concepts but which draw on other disciplines for their primary methodology. This includes implementation, scaling up, and economic and program evaluation. Second, applied research projects that primarily use systems methods. These projects draw on systems dynamics, social network analysis, causal loop diagrams and soft systems methodology. The work includes conceptualising and mapping elements of chronic disease issues; examining potential leverage points and testing intervention options and effects; identifying approaches to influence chronic disease that take account of complexity; and implementing changes to influence chronic disease prevention, reflecting on effects and revising strategies in collaboration with key stakeholders. The third model focuses on systems capacity building for researchers and policy partners which takes account of the differing levels of knowledge, experience and needs within each group. Details about the Partnership Centre’s activities and underlying model of knowledge mobilisation are available elsewhere.^63-66^

## Methods

This study was conducted as part of a larger mixed methods evaluation of the Prevention Centre.^64,66,67^ Previous interviews had been conducted with the Centre’s chief investigators and researchers, and a partnership engagement and impact survey had just been completed, so this was an opportunity to elicit policy-makers’ views and experiences in more depth, and to generate open dialogue in which any evaluative issues and ideas could be explored safely. A semi-structured interview-based study was chosen as the most effective approach.^68^

### Recruitment

Interviewees were identified from an online survey of stakeholders based in policy, program and services agencies (government, non-governmental organisations and health services delivery organisations) who had taken part in Prevention Centre workshops or events in the last 3 years. The survey was launched via the Prevention Centre website and Chronicle newsletter, then representatives from the Centre’s funding partners based in the agencies described above were asked by the Centre Director to circulate survey information to relevant people in their organisation. The survey was conducted between November 2017 and March 2018. Questions focused on stakeholder engagement with the Prevention Centre and how resources have been used. Seventy-nine people responded, and 53 respondents (67.1%) completed the whole survey.

At the end of the survey respondents were asked if they would be willing to take part in an interview exploring their views and experiences and, if so, to provide their name and contact details. Twenty-two respondents indicated that they were willing to participate in interviews. One of these was deemed ineligible because she had subsequently left her government position and taken a paid role with the Prevention Centre. The remaining 21 were sent invitations to take part in an interview and 18 (86%) agreed.

### Data Collection

Telephone interviews were held over a 5-week period by the lead author. Interviews were conducted fluidly as ‘conversations with purpose’ in which participants are treated as active, expert partners in the research.^68^ They ranged in length from 28-60 minutes, with an average duration of 40 minutes.

Interview questions covered 5 domains: policy-makers’ roles and context, their perceptions of the collaboration, what they were getting out of this approach (if anything), and their suggestions for the Centre’s future directions and improvement. Specific questions were asked about how the Centre’s model—including its goal to*“apply systems thinking to policy problems”* —was working in practice. Interviewees were encouraged to talk about their ‘real world’ experience and to define concepts in their own words. Audio recordings were professionally transcribed then checked for errors by the interviewer.

### The Sample

As Table shows, 17 of the 18 interviewees were employed in health policy or service delivery agencies, and one was employed in a preventive health role in local government. They were all involved in the development, implementation and/or evaluation of public health policies or programs. Many also had some responsibility for capacity development in their agencies. Participants spanned a wide range of positions both geographically and in terms of role focus and seniority. Nine worked in organisations that were formal funding partners in the Prevention Centre (referred to as *Centre funders* in the table), and seven worked in organisations that either had contributed funds to specific projects or pieces of work or were non-funders. Twelve (⅔) were female and 6 were male.

**Table:** Characteristics of Participating Stakeholders

**I**D	**Type of Organisation**	Role Level*	**State**	**Organisational Funder Status**
1	Health ministry/dept	Senior manager	NSW	Centre funder
2	Health ministry/dept	Senior manager	NSW	Centre funder
3	Local health organisation	Senior manager	NSW	Non-funding
4	Local health organisation	Non-managerial	NSW	Non-funding
5	Health ministry/dept	Senior manager	ACT	Centre funder
6	Health ministry/dept	Manager	ACT	Centre funder
7	Health ministry/dept	Non-managerial	ACT	Centre funder
8	Health ministry/dept	Non-managerial	ACT	Centre funder
9	Local health organisation	Manager	VIC	Non-funder
10	Health ministry/dept	Non-managerial	VIC	Centre funder
11	Local government	Non-managerial	VIC	Project funder
12	Health ministry/dept	Senior manager	TAS	Non-funding
13	Health ministry/dept	Manager	TAS	Non-funding
14	Health ministry/dept	Non-managerial	TAS	Non-funding
15	Health ministry/dept	Senior manager	QLD	Centre funder
16	Health ministry/dept	Manager	QLD	Centre funder
17	Health ministry/dept	Senior manager	WA	Non-funding
18	Health ministry/dept	Manager	SA	Project funder

Abbreviations: NSW, New South Wales; ACT, Australian Capital Territory; VIC, Victoria; TAS, Tasmania; QLD, Queensland; WA, Western Australia; SA, South Australia. * The distinctions between role levels is based on interviewees’ description of their position. They are approximate and not necessarily comparable across jurisdictions due to different organisational structures.

### Analysis

Data was analysed thematically by identifying the variety and prevalence of types of information that helped to answer (or provided additional insights into) the research questions.^69,70^ Two researchers independently coded some early transcripts and, with a third researcher, workshopped an initial coding frame which included organisational, substantive (descriptive) and conceptual categories.^71^ This coding frame was revised in relation to further data and as a result of ongoing discussions between the researchers, and was applied to all transcripts using NVivo 11.^72^ Codes were checked for accuracy and coherence by iteratively reviewing the range of data within them and their relationships to each other (for example, should a code be broadened, or integrated with another code, to better reflect the data?). This review considered how effectively each code was able to capture relevant data in individual transcripts and across the whole data set. As they developed, core themes were critically reviewed against examples of discrepant data to ensure the range of variation, and any counter-examples or contradictions were captured. Analysis also drew on the running memo kept by the lead researcher while conducting the interviews which included conceptual categories, questions and analytical ideas.^73^

This work was inductive (ie, there was no predetermined analytical framework) but it was interpreted through the lens of literature on knowledge mobilisation and collaborative partnerships in health research.^12,13, 74-79^ The theoretical perspective was underpinned by a ‘realist’^80^ or ‘contextualist’^70^ view of the data as representing individuals’ intersubjective meanings within a socially and materially structured (real) environment. This means that we can never fully know the ‘truth’ of a situation, but we can get closer to it through methods such as listening to, and triangulating, the views of people who experience it.^81^

Draft results were reviewed by all authors and revised through discussion. The diverse authorship group was well positioned to critique the interpretations given that one is an educator in systems thinking, one is a qualitative researcher with no systems background, and two are policy-makers with different views about the practical utility of systems thinking and the extent to which it offers a new way of tackling policy problems.

## Results

###  1. Why Did Policy-Makers Choose to Participate in a Systems-Focused Collaboration?

The most enthusiastic accounts of why busy policy-makers were involved with the Centre focused on its facilitation of cross-sector connectivity and collaborative work processes with highly respected researchers and counterparts in other policy jurisdictions.

The second most prominent reason for partnering with the Centre was its emphasis on systems thinking and the utility of this approach for addressing real world complexity. The combination of collaboration and systems thinking together promised innovative ways of tackling old problems, and good returns on investment of time and resources in the form of anticipated improvements to prevention policy and practice:

“…*to work with and have the opportunity to connect to great thinkers and practitioners in this space is really going to have a beneficial impact, I think, on the work for us here in [name of state], and I’m sure in other jurisdictions around Australia. I think that shows great promise for a sustainable way forward for tackling chronic conditions.”*

Many interviewees commented that the Centre model was delivering useful information, resources, tools and methods, and was increasing their own capacity and the capacity of the prevention workforce more broadly. These early outcomes, including policy impacts, are described in section 4 of the results.

### 2. How Are Policy Partners Experiencing Systems Thinking?

Of all the topics discussed in the interviews, systems thinking triggered the most polarised, passionate and ambivalent views. All interviewees agreed that systems thinking is both challenging and has some potential merit, but its value for prevention policy was contested.

At the most positive end of the spectrum, policy-makers talked about the energising effects of systems thinking on themselves and, often, their colleagues; and its tremendous potential for evidence-informed policy change. These stakeholders tended to be highly engaged in one or more programs of activity and were either applying systems-informed tools in their work or had colleagues who were doing so. Some of this group had a pre-existing interest in systems thinking and this was the ‘lure’ that triggered (and, in part, maintained) their engagement with the Centre. Others were introduced to systems thinking via by attending a Prevention Centre event, encountering a resource or hearing about it as part of a conference presentation. This group was excited by the fit with their work and chose to investigate the Centre’s work further which, in many cases had evolved into project partnerships:

“*I think our whole interest and energy in systems approaches really got inspired because of the Centre. We were hearing about this stuff but they came along and supercharged it. Or, even just, because they started talking about it and creating some opportunities, and providing some support, and so that means that we were able to, yeah, basically put it into action.”*

These policy-makers were motivated by the innovative prospects of systems thinking:

“*What’s really incredibly positive for me is the Centre is particularly committed to taking a systems approach to chronic disease prevention…. Not trying to duplicate the work of others but to step into areas which are new and emerging…. That’s the thing that really excites me, because I’ve spent some years in it and believe in it even though I don’t have the same depth or breadth of understanding in that intellectual research sense in my own practice experience.*”

However, not all partners were systems enthusiasts. Some policy funders said they struggled with systems thinking in the Centre’s formative months when investigators and lead researchers were trying to articulate the Centre’s ethos and identify shared goals. Several talked about the *“painful discussions”* led by *“evangelical researchers”* that slowed progress and threatened to alienate policy-makers. At these early meetings, project ideas that were not explicitly informed by systems science tended to be dismissed, *“[It felt like] if you weren’t taking a systems approach it wasn’t worth doing it.”*

There was general agreement that this developmental phase had passed. Some questioned the extent to which these deliberations had progressed their understanding or the Centre’s agenda, but others felt it had been necessary and may have resulted in greater cohesiveness. One example of this was that, over time, outputs from diverse projects were perceived as more consistent in their use of systems thinking. Equally, greater coherence may reflect evolution of the Centre’s identity, structure and processes, including allocation of resources, establishing a coordinating team, a more sophisticated communications strategy and the development of positive working relationships among heterogeneous partners.

Concern about definitional clarity seemed to affect those who were actively using systems approaches as well as the sceptics. Some partners with health promotion backgrounds saw systems thinking as a complement to their ecologically-orientated worldviews, while others were inclined to question the distinction between these perspectives:

“… *for a health promotion person, you see, I think every time I go to a systems session, ‘Is this new?’ I think it’s important and I agree with it but I feel like I’ve been doing systems thinking for my whole career, but probably not in the rigorous way in which they are now applying it. But myself, and my other older colleagues certainly feel that it’s not new, that it’s just re-named.”*

Even some of those who asserted that systems thinking is more than rebranded health promotion were unable to articulate the distinction:

“*I’ve often asked academics in this area, ‘What’s the difference between systems practice and ecological practice?’ … It’s taken me a while to actually start to understand, yes, there is a little difference. I don’t know that I could give a good explanation of what that actually is, but I’m becoming increasingly more aware that there is some level of difference.”*

Questions about how to define systems thinking impacted perceptions of its accessibility and usefulness. For example, one policy-maker who identified as a systems enthusiast explained that he did not recommend the Centre’s systems resources to colleagues for fear of *“scaring them off.”* While another expressed concern that her colleagues were taking ideas from the Centre back to their sectors because *“… it’s very easy to be confused and hopeless in systems theory.”* This interviewee was the most critical of the Prevention Centre’s systems orientation, calling it a*“red herring,”* but as she explained her reservations it became apparent that she understood the Centre’s work purely in terms of hard systems (often viewed as a more mechanistic positivist-leaning approach) and, specifically, mathematical modelling rather than the general systems approach which incorporates soft and critical systems strategies (viewed as a more person-centred and constructivist).^21^ Ironically, others explained their buy-in to the Centre’s systems-informed work exclusively in terms of soft systems:

“*I was definitely giving clear approval for that [systems-focused] conversation to keep going because… we are really engaged with, ‘How do we support our communities to take greater control, support their capacity building for their own health and wellbeing?’”*

Given this contrast it is not surprising that some interviewees suggested that the Centre should examine how it communicates its systems orientation. One policy-maker argued that communications were skewed towards dynamic simulation modelling which was in danger of eclipsing other valuable systems-informed work conducted by the Centre and was also relatively costly for policy agencies. There was a suggestion that hard and soft approaches could be better integrated in some projects.

Policy-makers who saw less value in a systems approach were agnostic rather than oppositional. Most of these were senior managers who were co-funding and/or supervising policy staff working on systems-informed projects but had little direct involvement in project development or capacity building themselves. They saw themselves as pragmatic and were concerned that the academic/theoretical aspects of the Centre’s work (which at times seemed to be conflated with the wider literature on systems thinking) were getting in the way of policy utility:

“…*in some cases they have involved experts that have come from overseas. They talk about their research, how complex systems are, but when they get asked, “Okay, can you give some practice examples of how this has been applied in a way that’s useful for policy?” They say, “Oh, no, I can’t think of any.” To me that just seems like a lot of wheel spinning work. I just think that this whole research community that’s grown up around that and it’s sort of their main area of expertise, but it’s not really having much impact on policies.”*

An interviewee who worked closely with practitioners felt similarly:

“…*my colleagues are interested to know more about systems but… don’t see many implications on the practice level…. I think at the practice level people are more interested to know practical problem-solving methodological issues…. When I casually spoke with some of my colleagues, many of them are not really very clear about what actually the study of systems analysis can give us, [what is] the benefit… in terms of program design, implementation and stuff.”*

Thus, the concern was not that systems thinking intrinsically lacks value, but that the focus could be more practical and policy/practice orientated. Even some of the most enthusiastic proponents of the Centre’s systems approach saw a potential threat in getting bogged down in theory: *“…the thinking is the place you start but then you’ve got to move beyond that, otherwise you just end up with a big scribble and feeling overwhelmed.”*

Across the interviews there was general agreement that the focus should be not on documenting complex systems but on identifying ways to intervene in them. For example, this policy-maker was arguing against using a systems lens to map complexity:

“*We already know that! Telling treasury and finance and ministers how complex things are is actually not that useful…. [What we want to know is] how do you navigate the complexity to come up with what is the information that’s most helpful and needed?”*

Nevertheless, nearly everyone who talked about systems thinking was able to identify cases where its application had potential, or identified processes or tools that they had already found beneficial:

“*I’m amongst the sceptic group but I certainly see where it does make a lot of sense in dynamic system modelling. And that’s, particularly, an area where we can take inputs about population, prevalence of particular behaviors and add that to the effectiveness of intervention or multiple interventions and then try to model what’s happening. I think it’s a really important tool for thinking through, systematically, how we can potentially scale up interventions and what the impacts might be.”*

### 3. What Is Supporting Policy Partners to Engage With Systems Thinking?

Policy-makers identified 2 primary aspects of the Centre as corollaries to an effective systems approach for policy. First, cross-sector co-production was seen as the foundational mechanism for getting value from the Centre:

“*You talked about being motivated by new ideas and new methods. Researchers often think they’ve got new policy-relevant ideas and methods, but they often haven’t. What’s different about what’s happening here?”* [Interviewer].

*“It’s the co-creation…. The fact that we’ve been able to literally co-produce some of the work that’s been researched gives us a great opportunity to ‘walk beside and learn from’ in our own practice”* [Policy-maker].

It seemed that those who were not participating in co-produced project work experienced the Centre’s outputs as less useful. For example, this policy-maker explained that she is interested in systems thinking but finds some of the Centre’s outputs to be impractical and poorly attuned to her needs:

“*It’s still difficult to grab stuff that the Centre does, though, and translate it into our own context. Like, it’s not directly transferable and some of the research stuff that gets done is interesting, but it’s not necessarily going to push our agenda along…. It’s a bit hard to… get a handle on... it’s researchy , and it’s academic, and most of the time it’s not all that directly useful for policy-making.*”

Second, tailored capacity development and support. In this example a manager is explaining how his policy division worked with the Centre to advance their application of systems approaches which included tailored workshops and ongoing dialogue:

“*We’d already been talking and then we had the workshop and we’ve continued to talk. It’s helped us to develop a series of actions that we can continue to implement... helped us to work out an approach about going forward, and so that’s how it’s been really helpful. I mean, it’s not an easy thing to do, and we’re trying to reorient it a little bit to the way we approach different aspects of our work… and where there are leverage points in our context…. But we’re absolutely a lot further down that path now than we would have been if we hadn’t had that support.”*

Capacity development was also strongly supported by the Centre’s communications and resources which provided, *“a straightforward language to talk about what we are doing”* and enabled policy-makers to describe systems-informed policy work in lay language for funders, managers and the public.

Many policy-makers talked about learning as multi-dimensional so they often attributed their capacity development to multiple activities associated with the Centre including seminars and workshops, mentoring, the ‘community of practice’ network which facilitates dialogue with systems experts and with peers who are applying systems thinking in their own localities, and the experiential problem-solving involved in project work:

“*The community of practice has been a great avenue to just get together and learn from others and build my own systems skills…. [It] has been really valuable and helps to keep systems thinking and continuous learning on the agenda, and it’s a good place for me to refer others to…. [Also] I guess the workshops that I’ve attended and the projects that I’ve been involved with and the coaching and mentoring that I’ve received… has really built my capacity, and that’s enabled me to deliver some tangible outputs.”*

Two aspects of this learning stood out. First, that it was applied: *“… as someone who’s literate in some of these areas I’ve gained a lot and really learned a lot about system science and those sorts of approaches…. And I’ve learned by doing which I think is often the best way to do it.”*

And second, that it took place in a context of mutual learning and shared expertise. When asked if and how they had benefitted from the Centre, policy-makers frequently pointed out that they were also *contributing* to it in terms of expertise, resources and access to their jurisdictions: *“I think it is genuinely both ways. [We’re] giving them access to people and communities and organisations and practice to work with and research.”*

Many of these key mediators for engaging in systems thinking—mentoring and coaching opportunities, participation in applied co-production and linkage with the Centre’s networks—are resource intensive and have access limitations. In most cases, only policy-makers who were based in funding agencies or had project-specific arrangements to work with the Centre’s Systems Thinking and Capacity Building Manager could access tailored support and participation.

### 4. What Value Have Policy Partners Found in Systems Thinking?

Policy interviewees identified a range of beneficial outcomes from their engagement with the Centre which, in most cases, were seen to be informed by systems thinking. They included the use of research, ideas, tools and resources; the development of practical methodologies that were helping policy-makers to design, scale up, implement and evaluate complex policies and programs; and the use of innovative prevention narratives to communicate persuasively for community engagement and education, policy action and funding. These are described elsewhere.^66^

Policy-makers also talked about a conceptual shift that was affecting multiple aspects of their work. They identified systems thinking as a key contributor to this shift which was not about acquiring knowledge or influencing a discrete policy, but about an evolving systems perspective that is changing how they think and talk about health problems and contexts, policy goals and practices, and approaches to developing solutions:

“[*We’re] thinking about systems and just thinking about things differently. I think we’ve moved right along; it’s a more sophisticated understanding now…. And my feeling is that through the Centre we’re getting the understanding, the knowledge and developing some skills to help us to do that work. We could only get so far previously, but I think actually we’re going ahead in strides now.”*

New ideas, methods and tools had catalysed and equipped this change, but processes of engagement and collaboration (which build in research translation) were also crucial:

“*It’s the participation which gets you thinking about your practice, gets you thinking about different [policy] opportunities. I think because we’re involved in this work we don’t wait for some glossy two-pager because we co-created it and we’ve communicated about it as we go.”*

In some cases, policy-makers found that systems thinking provided a shared framework and language that was helping them to become conscious about, and embed, intuitive work practices:

“[*It]has been a really interesting process of seeing people really embrace systems thinking and what their role is in the system in a local context. But also, I’ve had a lot of people recognising what they’re doing... like some people have been doing systems thinking things always, but they haven’t necessarily had the systems thinking labels…. It’s definitely coming up in conversations. People are actively seeking how they can integrate it into their work.”*

Systems-focused tools and methodologies were also having policy impacts. For example, here, a local government officer explains how a system-level evaluation approach helped her team to revise the focus of their work and convince those in power to support it:

“… *one of the things that was really useful for me was the Measuring Impacting Systems workshop because I’m developing and finalising our monitoring and evaluation plan and livability plan at the moment so my colleague who’s our community planner came along to that workshop with me and we thought about how we’re using different measures… and we started to build some of those into our evaluation plan…. When we took our plan to council we decided to, instead of just going for the four years which is what’s required under the legislation, we actually took it up to them as a 12-year plan. So we got commitment from the council to endorse it as a 12-year plan…. We changed perceptions internally and with our council around what our expectation is around what we should be able to achieve.”*

Another account indicated that systems tools were informing policy funding:

“*I think it has the potential to influence [our investment strategy], absolutely. Absolutely. I can hear people talking about it already. So when we come to planning meetings about future investments, we hear people say, ‘The dynamic simulation modelling, that’s saying we need to invest more here…. We need to push it in that direction.’ It is actually being used in conversation.”*

Yet it seemed that, most often, the Prevention Centre was influencing overarching strategic direction or thinking across a program of work rather than being applied to discrete policies or programs:

“[*For] our executives… [systems thinking] helps them to focus on some of the more insidious [aspects of chronic disease] and the longer-term goals that we’re heading towards… It’s hard when you’ve got a senior role in government not to get bogged down in government process, [so] it keeps them going in the right direction and keeps them motivated and keeps them up-to-date with innovation and how we could be doing things much better on an operational level. Just the knowledge base and the more free and innovative thinking of some of my colleagues, it’s just really upskilled them.”*

## Discussion

All 18 health policy interviewees in this study described some engagement with systems thinking which, in most cases, was inspired or sustained by their connection with the Prevention Centre. Those who were most enthusiastic talked with excitement about the potential of system-informed research, tools and methods to change the prevention discourse and generate new ways of tackling old problems. Many were advocates for systems approaches in their workplaces and identified impacts on their work. Policy-makers who were less enthusiastic were agnostic rather than oppositional. These self-described sceptics focused on confusion about what systems science is and how it can be applied practically, including the need to address complexity rather than describe it (an argument supported by Holmes et al^48^). Some explained they had little interest in systems thinking per se but saw value in the more concrete methods and tools that it was producing. Most of more skeptical policy-makers were senior managers who had little direct involvement in the Centre’s project development or capacity building activities. Given the importance of senior managers in directing policy, additional strategies may be required to demonstrate the value of systems approaches for this group.

The variation in responses is not surprising given the paradigm shift that systems thinking represents, and the perceived lack of robust examples of systems approaches being applied to policy which undermines its value proposition (although we note there are examples, eg,^11,14,27,34,59,82-85^). People make sense of the world given what they know so, without a compelling rationale, we tend to hold on to established mental models and avoid the disruption of seeing the world in radical new ways.^86,87^

Nevertheless, systems-focused research, ideas, tools and resources were being used in policy and program work. This was contributing to the development of practical methodologies that were helping policy-makers to design, scale up, implement and evaluate complex policies and programs, and was shaping the use of innovative prevention narratives in policy communications. The literature supports the assertion made by several study participants that they had been doing systems-informed work for many years, often by another name eg,^35,37^. But most of these policy-makers found that working with the Prevention Centre helped them to consolidate, articulate and expand their experiential learning, and to test ways of applying it to specific problems. This included developing different ways of describing and measuring their work, and of engaging stakeholders in new initiatives.

Perhaps most importantly, despite the cogitative challenges mentioned above, systems thinking seemed to be helping policy partners to reconceptualise health problems and contexts, goals, potential policy solutions, and approaches to developing those solutions, including prevention risk factors, outcomes and indicators, measures and roles. In short, policy partners were changing how they think about prevention.

The policy impact literature tends to focus on instrumental (direct) use of research or, when it comes to conceptual impact, policy-makers’ acquisition of knowledge, ie, *changing what we know* . However, it may be that *changing how we think* offers the greater potential for policy impact because it transforms people’s mental models and the principles by which they carry out their work.^16,88^ For example, Hall^89^ distinguishes between 3 types of policy change: in first-order change the targets or settings of policy action are altered, in second-order change the techniques used to achieve policy goals are altered, and in third-order change entirely new thinking about a policy issue emerges resulting in changed policy goals, discourse, epistemologies, instruments and techniques. Such third-order change has the ability to foster radical new policies and programs.^89^

Exposure to systems thinking alone, however, cannot be regarded as the primary mechanism for this change. Policy-makers did not express excitement about abstract theories or principles, but about applying systems thinking to specific concerns in their local contexts. Their accounts focused on critical reflection, dialogue with experts, pragmatic capacity building and support, co-production and learning through doing. This is a reminder that theory and practice (like knowledge and practice^76^) are not dichotomies but have a dialectical relationship known as praxis.^90^ It seems that, for some policy-makers at least, the Prevention Centre is facilitating praxis via action learning^91^ which involves asking questions and trying out policy solutions collaboratively.^92^ Systems thinking is thereby integrated into real world action. This is in contrast to the more common tendency for researchers to give systems advice to policy-makers in the form of vague precepts or warnings about what not to do, and then leave them to get on with it.^45^

The term ‘capacity development’ seems too poorly defined to capture the synergistic learning and reflective thinking identified by the more engaged policy partners. They did not talk about *learning from* researchers and peers (or certainly not *only* learning from them) but about *thinking with them* . This process of thinking-together-in-practice exemplifies the shift from a knowledge transfer paradigm towards co-produced research^77^ that produces *“socially robust knowledge,”* ie, problem-solving knowledge that is generated in the context of application, drawing on multiple forms of expertise.^93^ Freebairn et al^83^ reached a similar conclusion in their study of policy-makers’ experience of dynamic simulation modelling, finding that co-production built trust in the model and its outputs. Such knowledge is more likely to be perceived as valid and useful—or, in Weiss and Bucuvalas’s terms, to pass the ‘truth tests’ *and* ‘utility tests’ that policy-makers apply to research^94^ —but, it is also able to challenge and change business-as-usual, potentially contributing to wider scale transformational change.^34,58^ However, building the foundation for this work comes at a cost. Co-production is challenging and time consuming,^95^ and depends on people, resources and processes that can build a culture of reflective learning, facilitate power sharing, and deal with the inevitable tensions that arise when groups with diverse epistemic traditions come together.^77,96^

Health policy-makers are pragmatic, increasingly driven by return-on-investment and skilled at maximising resources^97^ so their views about the potential of systems thinking, and their decision to dedicate substantial time and money to the Centre, indicate a strong belief in the ability to turn systems thinking into positive policy action. This is driven by knowledge that, at times, significant investment in business-as-usual interventions has not resulted in desired sustained change. Nevertheless, questions about how systems thinking can best add value to their work were very much alive for policy partners who were trying to reconcile new concepts with established professional knowledge and practices. There is more work to do in articulating system thinking and in demonstrating its policy utility,^29^ including developing practical tools and real-world case studies that show how systems approaches can impact outcomes. The more that policy-makers engage in these efforts as collaborators, peer-educators and role models, the more effective we are likely to be.

### Strengths and Limitations

The participants in this study were a self-selected sample of policy-makers who voluntarily contributed to a survey and interview and may therefore be especially ‘invested’ in the Prevention Centre and more likely to support its ethos. However, the breadth of different roles, geographical locations and levels of seniority in this sample do suggest that the Centre’s approach to systems thinking is working well for a relatively wide group of policy stakeholders. We do not know to what extent their views reflect those of other policy partners, or how policy-makers who have no connection with the Centre might best be engaged in systems thinking.

Findings from this study are not directly transferable to other settings; these experiences of engaging with systems thinking occurred within a context of structures, activities and relationships that are specific to the Prevention Centre. But we hope that the findings can provide some insights, ideas and encouragement for other systems-focused cross-sector research collaborations. For example, it seems likely that policy-makers in other jurisdictions will have related questions and qualms about systems thinking, and that the support mechanisms identified here—co-production, capacity building and the emphasis on applied, mutual learning targeting priorities in policy-makers’ own jurisdictions—could play an important role in other similar endeavors.

In the introduction we describe 3 overarching models by which systems thinking is tackled in the Centre’s work: (1) Scholarly research projects that apply a systems lens but draw on methodologies from other disciplines, (2) Applied research projects that are founded on systems concepts and methods, and (3) Systems capacity building. However, in our analysis we were unable to definitively identify the extent to which each study participant was engaged in one or more of these, so their different forms of engagement (or lack of engagement) with the Centre’s different approaches to systems thinking tend to be collapsed in the results. If we had spent more time unpacking how policy-makers’ views were linked to their experience of specific approaches it could have helped us better understand what was working for whom, and why. So, while this paper offers some useful insights into policy-makers’ engagement with systems thinking, it is somewhat generalised and could be strengthened using a more rigorous approach.

This paper focuses on interviews with policy-makers, so we cannot comment on how systems thinking is impacting researchers at the Centre, including the extent to which early- and mid-career researchers (many of whom will have bio-medical backgrounds) might be reconceptualising prevention and embracing new methods. Given that the Centre strives to build capacity and adapt reflexively, this is well worth investigating.

## Conclusion

These findings offer 3 lessons. First, researchers and policy-makers can put systems thinking into policy action as part of a research collaboration. We show how this has occurred within the Australian Prevention Partnership Centre and identify some continuing challenges. Second, knowledge processes may be more important than knowledge products. We found that policy-makers who were working with the Centre saw considerable value in mutual learning about, and practical application of, systems thinking, but this was served primarily by co-production and capacity building rather than ‘receiving’ information. Policy-makers’ participation in iterative processes of reflection and interaction helped orientate these projects to address real-world policy concerns and, in the words of one interviewee, *“surface the ‘so-what?’”* Third, policy-makers’ engagement with systems thinking can contribute productively to a range of outcomes. In this study outcomes included the development and use of research, ideas, tools, resources, practical methodologies and innovative prevention narratives. But, perhaps most importantly, policy partners were reconceptualising health problems and contexts, goals, indicators and policy solutions. They were changing how they think about prevention. We argue that this has the potential for far reaching policy impacts. Systems thinking is not a panacea for policy problems which are often entangled in complex social, economic, political and institutional contexts, but it does offer tools and strategies for better understanding these contexts and, potentially, for strengthening policies so that they are more inclusive, effective and resilient.^60^ The next challenge is to demonstrate how the use of system thinking in policy processes has enhanced effectiveness or other measurable outcomes such as policy acceptability or sustainability.

## Acknowledgements

Sincere thanks to the 18 pioneering policy-makers who kindly contributed to this study. Thanks also to Hayley Hughes who helped with the initial coding of the interview data, and Emma Slaytor and Andrew Wilson for their comments on the draft manuscript.

## Ethical issues

Ethical approval for this study was given by the Sax Institute, Ultimo, NSW, Australia, ref R20180430. All participants gave informed consent and were free to withdraw from the study at any time.

## Competing interests

Authors declare that they have no competing interests.

## Authors’ contributions

AH designed this study, collected and analysed the data and drafted the manuscript. KG, SD, and AM made substantial intellectual contributions to revisions of the manuscript.

## Authors’ affiliations

^1^The Australian Prevention Partnership Centre, Sydney, NSW, Australia. ^2^Sydney School of Public Health, University of Sydney, Sydney, NSW, Australia. ^3^Australia Public Health Services, Department of Health Tasmania, Hobart, TAS, Australia. ^4^The Systems School, Melbourne, VIC, Australia. ^5^Centre for Epidemiology and Evidence, NSW Ministry of Health, Sydney, NSW, Australia.

## Key messages

**Implications for policy makers**

- Policy-makers can make practical use of systems thinking, resulting in positive impacts on policy processes and expected longer-term impacts on preventive health.
- Some policy-makers who took part in a collaboration that uses systems thinking reported that it changed the research, ideas, tools and resources they were drawing on which impacted (a) the methods they were using to design, scale up, implement and evaluate policies, and (b) how they were talking about prevention in their own organisations and with stakeholders.
- Systems thinking offers new ways of conceptualising health problems and contexts which opens up innovative ways of working with communities and of tackling wicked policy problems.

**Implications for the public**

Systems thinking provides a different (and potentially better) way of understanding and tackling complex health problems like obesity. But systems thinking can be overwhelming and hard to use practically. An Australian partnership of policy-makers and researchers is trying to put systems thinking into action. Our research found that some of the policy-makers in this partnership were struggling to understand and use systems thinking, but most of them were using it in their work and starting to see real benefits. It seems that where the research and initial policy action was done collaboratively (ie, the partners made decisions and did the work together) policy-makers learnt a lot and found practical ways to put their learning into action. Pragmatic support strategies and the long time frame were probably important too. We hope this work will eventually change policies so that they are more effective in helping people to prevent avoidable health problems.
